# Nurses' knowledge, attitude, and practice toward the sexual health of breast cancer patients

**DOI:** 10.3389/fpubh.2025.1654268

**Published:** 2025-12-15

**Authors:** Yingjie Gong, Dan Zhao, Qiannan Ye, Xin Shao, Lingling Tang

**Affiliations:** 1 Oncology Department, Nanjing Luhe People's Hospital, Yangzhou University, Nanjing, Jiangsu, China; 2 Nursing Department, Nanjing Luhe People's Hospital, Yangzhou University, Nanjing, Jiangsu, China

**Keywords:** knowledge, attitude and practice, sexual health, breast cancer, cross-sectional study, structural equation modeling

## Abstract

**Background:**

This study explores the knowledge, attitude, and practice (KAP) regarding the sexual health of breast cancer patients among nurses in Nanjing, China.

**Methods:**

Conducted from June to July 2023 across six hospitals, this web-based cross-sectional study utilized a self-administered questionnaire, resulting in 252 valid responses, predominantly from female nurses (98.41%).

**Results:**

The mean scores indicated inadequate knowledge (8.82 ± 5.33), a positive attitude (38.43 ± 5.23), and inactive practice (27.75 ± 6.68) regarding patients' sexual health. Multivariate logistic regression identified that the lack of training on breast cancer sexual health was significantly associated with lower knowledge (OR = 0.31, *p* = 0.001), while job satisfaction correlated positively with knowledge levels (OR = 1.28, *p* = 0.018). Age (OR = 1.13, *p* = 0.001) and attitude (OR = 1.22, *p* < 0.001) were linked to higher levels of practice. Structural equation modeling revealed that knowledge significantly positively influenced attitude (*β* = 0.493, *p* < 0.001), and both knowledge and attitude directly affected practice (*β* = 0.563 and *β* = 0.897 respectively, *p* < 0.001).

**Conclusion:**

Overall, nurses demonstrated a need for improved knowledge and active practices concerning the sexual health of breast cancer patients.

## Introduction

Breast cancer represents a significant health burden in China, with approximately 357,200 new cases in 2022, ranking as the second most common cancer and fifth leading cause of cancer-related mortality among Chinese women (1). Beyond its physical impact, breast cancer treatment also substantially affects young women's physical appearance, sexual and reproductive functioning, and employment opportunities (2).

A balanced sexual life constitutes an integral component of overall well-being, but many breast cancer treatments, including radiotherapy and chemotherapy, and surgical interventions, can significantly impair patients' sexual quality of life, with approximately 60% of patients experiencing diminished sexual satisfaction following treatment (3). This disruption occurs primarily due to treatment-induced physiological changes such as hormonal fluctuations, vaginal dryness, pain during intercourse, reduced libido, and loss of breast sensation or physical disfigurement from surgeries like mastectomy or lumpectomy (4). These physiological factors, coupled with psychological sequelae including altered body image, anxiety, and depression, severely affect patients' sexual self-confidence, femininity, and intimacy with partners (5).

Despite these significant impacts, sexual health remains largely neglected in cancer care protocols in China, with effective communication between patients and doctors about sexuality being infrequent, despite 68% of breast cancer patients expressing a desire for information on this topic (6). Patients often hesitate to initiate these discussions, highlighting the crucial role nurses play in initiating open dialogues and providing empathetic, professional guidance on sexual health (7). Therefore, comprehensive training and positive attitudes among nurses are essential to effectively support breast cancer patients in addressing their sexual well-being. The Knowledge-Attitude-Practice (KAP) model represents one of the most widely employed theoretical frameworks for studying the behaviors of healthcare professionals. In the realm of health literacy, it operates on the premise that knowledge functions as a catalyst, positively shaping attitude, which in turn guides practice. According to the KAP theory, the process of altering human behavior unfolds across three distinct stages: the acquisition of knowledge, the cultivation of attitude and beliefs, and the subsequent formation of practice and behaviors (8). Within clinical nursing practice, nurses' knowledge and attitudes directly influence their interactions with patients. Specifically, nurses equipped with comprehensive knowledge and positive attitudes toward sexual health are more likely to proactively address patients' concerns, provide evidence-based information, and foster open communication, thereby improving patients' overall sexual health outcomes (9). In the context of breast cancer sexual health, nurses play a crucial role by disseminating accurate information and addressing patients' misconceptions and concerns.

Globally, studies on breast cancer patients' sexual health have primarily focused on the psychological distress, body image concerns, relationship difficulties, and barriers to communication between patients and healthcare providers (3, 10). However, few have specifically examined nurses' competencies or roles in addressing these issues, particularly within the Chinese healthcare context. Given the conservative attitudes toward sexual health discussions in Chinese culture, nurses may face greater challenges in providing effective patient support. It is essential to explore Chinese nurses' knowledge, attitudes, and practices regarding breast cancer patients' sexual health. Therefore, this study aimed to investigate the KAP toward the sexual health of breast cancer patients among nurses.

## Materials and methods

### Study design and participants

This web-based cross-sectional study was conducted between June 2023 and July 2023, across six hospitals of Nanjing, China (Nanjing Luhe People's Hospital, Nanjing Luhe Hospital of Chinese Medicine, Nanjing Jiangbei Hospital, Nanjing Gaochun People's Hospital, Zhongda Hospital Southeast University (Jiangbei) and Nanjing Pukou People's Hospital) and nurses were enrolled. Inclusion criteria were: (1) nursing staff from oncology, radiotherapy, general surgery (including breast surgery), thyroid and breast surgery, and breast surgery departments, (2) aged between 18 and 60 years. Exclusion criteria were: (1) do not agree to participate in this study. This study was approved by the Medical Ethics Committee of Nanjing Luhe People's Hospital (LHLL2021004) and informed consent was obtained from the participants.

### Questionnaire

This questionnaire was developed based on the relevant literature as the foundational framework (11). Subsequently, the initial design underwent refinements reviewed by six associate senior experts. A pilot test, involving 29 samples, was conducted to assess the questionnaire's reliability, yielding a reliability coefficient (Cronbach's α =0.883).

The final questionnaire in Chinese (a version translated into English was attached as an Appendix) composed four distinct sections. The first section covered demographic characteristics such as age, gender, education, nature of employing institution, job title, department, clinical work experience, involvement with breast cancer patients, relevant training received, and job satisfaction. Job satisfaction was evaluated by 0–10 points, 0 points indicated very dissatisfied, 10 points indicated very satisfied. The knowledge dimension comprised a total of 11 questions, with a scoring system assigning 2 points for well known, 1 point for partly known, and 0 points for unknown, resulting in a score range of 0–22. The attitude dimension comprised 10 questions and employed a five-point Likert scale, ranging from “strongly agree” (5 points) to “strongly disagree” (1 point), yielding a score range of 10–50. The practice dimension consisted of 8 questions, also employing a five-point Likert scale, spanning from “always/very consistent” (5 points) to “never/very inconsistent” (1 point), with a score range of 8–40.

### Quality control and distribution process

An electronic questionnaire was created using the online platform Sojump, with a corresponding QR code supplied for convenient access to the survey. Participants had the option to either scan the QR code via WeChat or follow the provided link to access and complete the questionnaire. To uphold data quality and ensure comprehensive responses, a one-submission-per-IP address restriction was implemented, and all questionnaire items were designated as mandatory. Participants were guaranteed anonymity during the survey process.

The research team, which included three investigators trained as research assistants responsible for questionnaire promotion and distribution, diligently examined all submissions for completeness, internal consistency, and logical coherence. Additionally, questionnaires completed in under 60 s, those containing logical errors, or those with uniform responses across all items were categorized as invalid.

### Statistical analysis

The sample size was determined to be 5–10 times the number of questionnaire items (12), which, in this case, was 29 independent variables. Consequently, the minimum required sample size was calculated to be 145. To account for a potential 5% invalidity rate among survey questionnaires, a minimum of 153 participants were needed to ensure adequate valid responses.

Statistical analysis was conducted using SPSS 26.0 (IBM Corp., Armonk, N.Y., USA). Quantitative variables were described using mean ± standard deviation (SD), and between-group comparisons were performed using *t*-tests or analysis of variance (ANOVA). Categorical variables were presented as *n* (%). Spearman correlation analysis was employed to assess the correlations between knowledge, attitude, and practice (KAP) scores. In multivariate logistic regression analysis, 70% score distribution of the total score was used as the cut-off value (13). Variables in univariate logistic regression analysis with *p* < 0.05 were enrolled in multivariate logistic regression analysis. Hypotheses were validated using structural equation modeling (SEM): (1) knowledge had impacts on attitude; (2) knowledge had impacts on practice; (3) attitude had impacts on practice. Two-sided *p* < 0.05 were considered statistically significant in this study.

## Results

Initially, a total of 270 responses were collected, and these included two cases with a response time of less than 60 s, two cases of disagreement with informed consent, one case with an abnormal age (126 years old) and one case with a missing age, four cases of irrational responses, and 11 cases of questionnaires from doctors. Ultimately, 252 cases were retained, with a validity rate of 93.33%. Among them, 248 (98.41%) were female, 132 (52.38%) aged 21–30 years, 196 (77.78%) held at least a Bachelor's degree, 223 (88.49%) were affiliated with Public Level 2 or above institutions, and 151 (59.92%) had a junior title (59.92%). Overall, in terms of departments, General Surgery/Thyroid and Breast Surgery/Breast Surgery constituted the largest group (121, 48.02%). 218 (86.51%) reported an experience in managing breast cancer patients and 169 (67.06%) had received training on breast cancer patient sexual health. The average job satisfaction score was 8.43 ± 1.72 (Table 1).

**Table 1:** Demographic information.

**Variables**	***N* (%)**	**Knowledge, mean ±SD**	***p***	**Attitude, mean ±SD**	***p***	**Practice, mean ±SD**	***p***
*N*	252						
Total score		8.82 ± 5.33		38.43 ± 5.23		27.75 ± 6.68	
**Gender**							
Male	4(1.59)	14.25 ± 4.57	0.650	38 ± 4.32	0.838	28.25 ± 2.87	0.970
Female	248 (98.41)	13.16 ± 5.35		38.44 ± 5.25		27.75 ± 6.72	
**Age (years)**							
21–30	132 (52.38)	12.76 ± 5.09	0.328	38.69 ± 5.37	0.756	28.91 ± 6.32	0.010
31–40	105 (41.67)	13.67 ± 5.66		38.06 ± 5.15		26.69 ± 6.93	
≥41	15 (5.95)	13.33 ± 5.05		38.73 ± 4.65		25 ± 6.38	
**Education**							
High school/college/associate degree	56 (22.22)	12.62 ± 5.46	0.325	40 ± 5.56	0.021	29.82 ± 5.90	0.010
Bachelor's degree and above	196 (77.78)	13.33 ± 5.30		37.98 ± 5.06		27.16 ± 6.78	
**Nature of employing institution**							
Other	29 (11.51)	11.55 ± 5.71	0.105	36.65 ± 5.05	0.027	26.55 ± 6.16	0.291
Secondary or above	223 (88.49)	13.39 ± 5.26		38.66 ± 5.22		27.91 ± 6.74	
**Job title**							
Junior	151 (59.92)	12.68 ± 5.22	0.071	38.39 ± 5.33	0.925	28.84 ± 6.44	0.006
Intermediate	90 (35.71)	13.75 ± 5.59		38.45 ± 5.16		26.1 ± 6.88	
Associate/senior	11 (4.37)	15.27 ± 3.95		38.90 ± 4.74		26.45 ± 5.69	
**Department**							
Oncology	105 (41.67)	12.73 ± 5.15	0.400	38.75 ± 5.59	0.258	27.54 ± 6.75	0.794
General surgery/thyroid and breast surgery/breast surgery	121 (48.02)	13.70 ± 5.42		37.96 ± 4.86		28.02 ± 6.38	
Radiotherapy	26 (10.32)	12.53 ± 5.61		39.34 ± 5.38		27.38 ± 7.89	
**Clinical work experience (years)**							
< 3 years	83 (32.94)	13.76 ± 4.91	0.023	38.68 ± 5.15	0.505	30.66 ± 4.89	0.001
≥3 but < 5 years	75 (29.76)	11.60 ± 4.96		39.21 ± 5.19		28.93 ± 6.08	
≥5 but < 10 years	94 (37.3)	12.37 ± 5.81		37.54 ± 5.43		27.06 ± 7.41	
≥10 years	83(32.94)	14.06 ± 5.13		38.74 ± 5.11		26.35 ± 6.63	
**Involvement with breast cancer patients**							
Yes	218 (86.51)	13.36 ± 5.30	0.204	38.39 ± 5.22	0.657	27.59 ± 6.80	0.287
No	34 (13.49)	11.97 ± 5.48		38.67 ± 5.34		28.82 ± 5.85	
**Received training on breast cancer patient sexual health**							
Yes	169 (67.06)	14.18 ± 5.39	< 0.001	38.78 ± 5.19	0.176	28.64 ± 6.77	0.001
No	83 (32.94)	11.13 ± 4.61		37.72 ± 5.27		25.95 ± 6.15	
**Job satisfaction (score) (M** **±SD)**	8.43 ± 1.72						

The mean knowledge, attitude and practice scores were 8.82 ± 5.33 (possible range: 0–22), 38.43 ± 5.23 (possible range: 10–50), and 27.75 ± 6.68 (possible range: 8–40), respectively. Participants in the age group of 21–30 years were more likely to have a proactive practice (*p* = 0.010). Individuals with Bachelor's degrees and above were more likely to exhibit positive attitude (*p* = 0.021) and proactive practice (*p* = 0.010) toward breast cancer care. Those working in public 2 or above were more likely to have positive attitude (*p* = 0.027). Clinical work experience also played a significant role, with participants having less than 3 years of work experience being more likely to have proactive practice (*p* = 0.001), but those with more than 10 years of work experience being more likely to have adequate knowledge (*p* = 0.023) and proactive practice (*p* = 0.001). Furthermore, those who received training on breast cancer patient sexual health were more likely to have adequate knowledge (*p* < 0.001) and proactive practice (*p* = 0.001; Table 1).

The distribution of knowledge dimensions revealed that the highest proportion of participants (46.43%) were familiar with the recommendation for physical barrier contraception over hormonal methods in breast cancer patients (K5), followed by awareness of the benefits of moderate sexual activity paired with contraceptive guidance (K4, 43.65%), and recognition of treatment-related factors (endocrine therapy, body image issues, anxiety/depression) impacting sexual health (K3, 39.29%; Supplementary Table 1).

A consensus emerged in the attitude dimension, with 87.7% of respondents (48.02% strongly agreeing, 39.68% agreeing) affirming the significance of sexual health to overall well-being (A1). Most participants supported societal guidance on sexual health aligned with moral beliefs (A2, 67.86% strongly agreed, 23.81% agreed) and emphasized the necessity for healthcare providers to address sexual health during follow-ups (A4, 50.4% strongly agreed, 36.51% agreed). Additionally, 84.12% endorsed using interviews/questionnaires to assess sexual health (A5, 35.71% strongly agreed, 48.41% agreed), while 88.89% highlighted the importance of post-treatment sexual health education (A6, 51.19% strongly agreed, 37.7% agreed; Supplementary Table 2).

In practice dimension, 27.78% of healthcare providers frequently assessed patients' sexual health via interviews/questionnaires (P1), while 25.4% routinely delivered sexual health education (P2). Notably, 78.97% provided preoperative/postoperative breast reconstruction guidance to patients with breast loss (P4). Additionally, 36.9% engaged patients' spouses in addressing sexual health concerns (P6), and 37.7% integrated sexual health considerations into individualized treatment plans (P7). A majority (64.68%) actively updated their knowledge on cancer-related sexual health research (P8; Supplementary Table 3).

Correlation analysis showed that significant positive correlations were observed between knowledge and attitude (r = 0.3035, *p* < 0.001), knowledge and practice (*r* = 0.5078, *p* < 0.001), as well as attitude and practice (*r* = 0.3808, *p* = 0.004; Supplementary Table 4).

Multivariate logistic regression analysis showed that the absence of training on breast cancer patient sexual health (OR = 0.31, 95% CI: 0.15–0.64, *p* = 0.001) and job satisfaction (OR = 1.28, 95% CI: 1.04–1.57, *p* = 0.018) were independently associated with adequate Knowledge. Meanwhile, attitude (OR = 1.22, 95% CI: 1.13–1.32, *p* < 0.001) and age (OR = 1.13, 95% CI: 1.05–1.22, *p* = 0.001) were independently associated with positive practice (Tables 2, 3).

**Table 2:** Univariate analysis of knowledge and practice.

**Variables**	**Knowledge**		**Practice**	
	**OR (95% CI)**	***p***	**OR (95% CI)**	***p***
**Knowledge**			1.22 (1.15–1.30)	< 0.001
Attitude			1.19 (1.13–1.26)	< 0.001
**Gender**				
Male	REF.		(empty)	
Female	0.45 (0.06–3.25)	0.429	(omitted)	
**Age (years)**				
21–30	REF.		REF.	
31–40	1.77 (1.01–3.08)	0.043	0.56 (0.31–1.00)	0.051
≥41	1.44 (0.45–4.51)	0.531	0.27 (0.06–1.28)	0.101
**Education**				
High school/college/associate degree	REF.		REF.	
Bachelor's degree and above	1.48 (0.75–2.92)	0.247	0.50 (0.27–0.93)	0.031
**Nature of employing unit**				
Other	REF.		REF.	
Public level 2 or above	1.22 (0.51–2.90)	0.643	1.10 (0.46–2.61)	0.823
**Job title**				
Junior	REF.		REF.	
Intermediate	1.89 (1.07–3.31)	0.026	0.52 (0.29–0.96)	0.037
Associate/senior	3.56 (1.03–12.3)	0.045	0.18 (0.02–1.48)	0.112
**Department**				
Oncology	REF.		REF.	
General surgery/thyroid and breast Surgery/breast surgery	1.59 (0.89–2.82)	0.112	0.81 (0.46–1.45)	0.493
Radiotherapy	1.52 (0.61–3.83)	0.365	0.96 (0.38–2.45)	0.948
**Clinical work experience (years)**				
< 3 years	REF.		REF.	
≥3 but < 5 years	0.69 (0.24–1.95)	0.488	1.06 (0.43–2.61)	0.898
≥5 but < 10 years	0.93 (0.41–2.08)	0.87	0.67 (0.31–1.44)	0.313
≥10 years	1.74 (0.83–3.66)	0.142	0.44 (0.20–0.93)	0.034
**Involvement with breast cancer patients**				
Yes	REF.		REF.	
No	0.63 (0.27–1.47)	0.294	1.00 (0.45–2.21)	0.995
**Received training on breast cancer patient sexual health**				
Yes	REF.		REF.	
No	0.25 (0.12–0.51)	< 0.001	0.41 (0.21–0.78)	0.007
**Job satisfaction (score) (M** **±SD)**	1.35 (1.11–1.64)	0.002	1.49 (1.20–1.86)	< 0.001

**Table 3:** Multivariate analysis of knowledge and practice.

**Variables**	**Knowledge**		**Practice**	
	**OR (95% CI)**	***p***	**OR (95% CI)**	***p***
**Knowledge**				
Attitude			1.22 (1.13–1.32)	< 0.001
**Age (years)**			1.13 (1.05–1.22)	0.001
21–30	REF.			
31–40	1.00 (0.39–2.53)	0.988		
≥41	0.52 (0.10–2.49)	0.415		
**Education**				
High school/college/associate degree			REF.	
Bachelor's degree and above			0.53 (0.19–1.41)	0.207
**Job title**				
Junior	REF.		REF.	
Intermediate	2.00 (0.78–5.10)	0.143	0.44 (0.12–1.52)	0.196
Associate/senior	3.75 (0.75–18.7)	0.106	0.11 (0.00–1.38)	0.088
**Clinical work experience (years)**				
< 3 years			REF.	
≥3 but < 5 years			2.21 (0.66–7.33)	0.193
≥5 but < 10 years			1.40 (0.45–4.33)	0.555
≥10 years			0.98 (0.21–4.63)	0.987
**Received training on breast cancer patient sexual health**				
Yes	REF.		REF.	
No	0.31 (0.15–0.64)	0.001	0.62 (0.28–1.40)	0.256
**Job satisfaction (score) (M** **±SD)**	1.28 (1.04–1.57)	0.018	1.21 (0.94–1.56)	0.126

**Figure 1:**
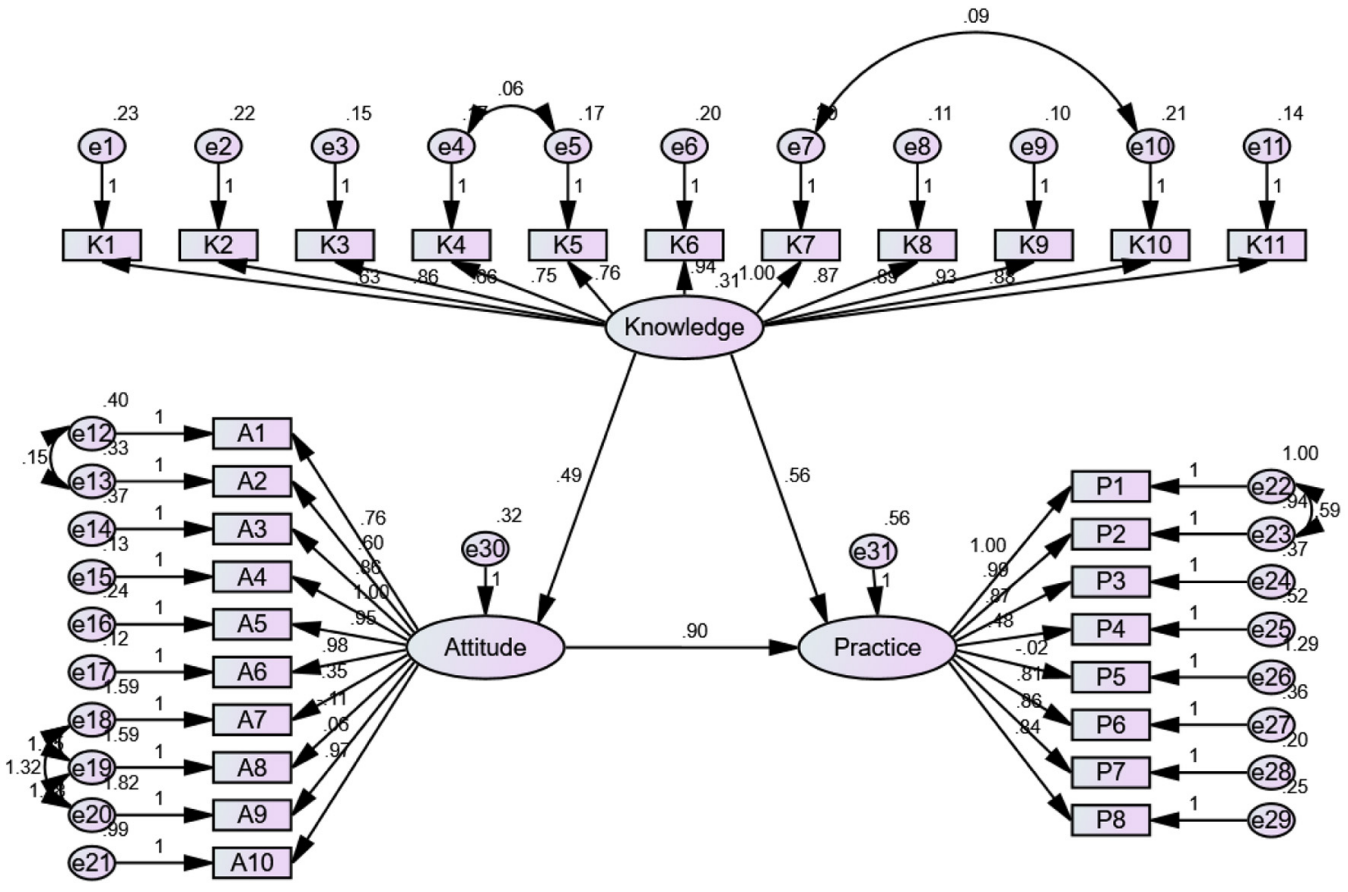
SEM model: the SEM included three latent variables, knowledge, attitude, and practice, and their corresponding observed indicators (K1–K11, A1–A10, and P1–P8, respectively). All observed variables loaded significantly onto their respective latent constructs. The model also includes measurement error terms for each observed variable (e1–e31), as well as several covariances among specific error terms within the knowledge dimension (e.g., between e4 and e5, e7 and e8, and e8 and e9), indicating shared variance among certain items. The structural paths show that knowledge has a direct positive effect on attitude (*β* = 0.493), and Practice (*β* = 0.563), while attitude exerts the strongest direct influence on Practice (*β* = 0.897). Overall, the SEM visually demonstrates the interrelationships among the three KAP components and supports the theoretical framework tested in this study.

The SEM included three latent variables, knowledge, attitude, and practice, and their corresponding observed indicators (K1–K11, A1–A10, and P1–P8, respectively). All observed variables loaded significantly onto their respective latent constructs. The model also includes measurement error terms for each observed variable (e1–e31), as well as several covariances among specific error terms within the Knowledge dimension (e.g., between e4 and e5, e7 and e8, and e8 and e9), indicating shared variance among certain items. The structural paths show that knowledge has a direct positive effect on attitude (*β* = 0.493) and practice (*β* = 0.563), while attitude exerts the strongest direct influence on practice (*β* = 0.897). Overall, the SEM visually demonstrates the interrelationships among the three KAP components and supports the theoretical framework tested in this study (Figure 1 and Supplementary Table 5). The SEM demonstrate a highly favorable model fit indices, suggesting a well-fitting model (Supplementary Table 6).

## Discussion

Nurses demonstrated inadequate knowledge, positive attitude, and inactive practice toward the sexual health of breast cancer patients. To improve patient care in this regard, healthcare institutions should provide specialized training, create a supportive environment, promote positive attitude, offer age-specific training, encourage interdisciplinary collaboration, and conduct regular assessments for nurses.

Previous international studies on breast cancer patients' sexual health have indicated that nurses commonly exhibit knowledge gaps and inconsistent clinical practices in addressing sexual concerns, partly due to insufficient training and discomfort with the topic (14, 15). In China, limited research has revealed similar findings, with nurses often reluctant to discuss sexual health issues openly due to traditional cultural norms and embarrassment surrounding sexuality (16). Such studies emphasize the need for targeted educational interventions and culturally sensitive training tailored to Chinese nurses. Our study contributes to this field by specifically examining Chinese nurses' KAP toward breast cancer patients' sexual health, thereby highlighting specific areas for intervention within the Chinese healthcare context. The regression analysis identified several independent predictors influencing nurses' knowledge, attitudes, and practices. Specifically, nurses with at least a Bachelor's degree demonstrated more positive attitudes and proactive practices, suggesting the value of integrating sexual health education into healthcare curricula (13). Clinical experience demonstrated a nuanced impact, with less-experienced professionals displaying greater proactive practice, while their more experienced counterparts exhibit higher knowledge levels and proactive practice, emphasizing the potential benefits of collaboration between these groups. Moreover, specialized training and higher job satisfaction were positively associated with nurses' knowledge, emphasizing the need for targeted education and a supportive working environment. Younger age and positive attitudes were independently linked to proactive practice, highlighting the potential for leveraging younger nurses' innovative perspectives and enhancing attitudes through comprehensive educational initiatives to ultimately improve patient care (17).

The survey results highlight that a majority of respondents within the healthcare community acknowledge the significance of sexual health in breast cancer care, recognizing its impact on overall human health and the quality of life of breast cancer survivors (18). However, there exists a divergence of opinions among some respondents toward whether sexual behavior should be considered a private matter, indicating a need for ongoing discussion and consensus-building within the healthcare community. To bridge these gaps, it is imperative to facilitate an open dialogue and establish guidelines for approaching this sensitive topic when communicating with patients (19). Furthermore, the study indicates variations in healthcare providers' knowledge levels across different dimensions of sexual health in breast cancer care. While some aspects, such as the use of physical barrier contraception methods and the importance of moderate sexual activity, are well-understood, uncertainty prevails in areas like the use of assessment tools and the potential impact of certain medications on sexual function. This underscores the importance of targeted training and education to ensure that healthcare providers are equipped to comprehensively address sexual health concerns with breast cancer patients. The findings also reveal that while some healthcare providers do employ methods to assess patients' sexual health and provide relevant education, there is room for improvement in the practice. Only a minority consistently inform patients about potential impacts on sexual function and offer guidance within treatment plans. Additionally, a small percentage proactively initiate discussions about sexual health, even when patients haven't broached the topic themselves. This highlights an opportunity for the development of training programs and guidelines aimed at encouraging healthcare providers to take a more proactive role in addressing sexual health concerns with breast cancer patients (20).

The correlation analysis unveiled robust positive associations between knowledge and attitude, knowledge and practice, as well as attitude and practice within the cohort of healthcare providers. This implies that individuals with elevated levels of knowledge tend to manifest more favorable attitude and exhibit more proactive practice in the context of breast cancer care. Hence, the promotion of knowledge acquisition among healthcare providers can potentially trigger a ripple effect, subsequently enhancing their attitude and practice, ultimately translating into improved care outcomes for breast cancer patients (21).

The SEM results provide a comprehensive perspective on the intricate relationships among knowledge, attitude, and practice in the context of breast cancer care. Notably, it was observed that knowledge exerts a direct and statistically significant influence on attitude, while both knowledge and attitude independently wield direct and significant effects on practice. In essence, the SEM findings affirm that knowledge serves as a linchpin for facilitating comprehensive sexual health care for breast cancer patients, highlighting its overarching importance in shaping healthcare providers' attitude and practice in this critical domain (22).

This study had several limitations. Firstly, this study's reliance on a web-based self-administered questionnaire raises concerns about potential sampling bias, as it may disproportionately include participants with better internet access and technological proficiency. Secondly, self-reported data collection introduces the possibility of social desirability bias, potentially leading participants to provide responses they deem socially acceptable rather than reflecting their true KAP. Lastly, the cross-sectional design employed restricts the ability to establish causal relationships or track changes in nurses' knowledge, attitude, and practice over time.

In conclusion, nurses had inadequate knowledge, positive attitude, and inactive practice toward the sexual health of breast cancer patients. Given the influence of Chinese cultural sensitivities on attitudes toward sexuality, targeted and culturally adapted training programs are crucial. Healthcare institutions should therefore prioritize comprehensive sexual health training, enhance supportive environments, and foster positive attitudes among nurses, ultimately leading to improved patient outcomes.

## Data Availability

The original contributions presented in the study are included in this article/Supplementary material, further inquiries can be directed to the corresponding author.
