# Supplementary material for: Nurses' knowledge, attitude, and practice toward the sexual health of breast cancer patients
Source: Front Public Health. 2025 Dec 15;13:1654268. doi: 10.3389/fpubh.2025.1654268 (PMC12745200; doi:10.3389/fpubh.2025.1654268)
Supplement: Supplementary file 1 [file Supplementary_file_1.docx]

| Questionnaire No. : | | | |
| --- | --- | --- | --- |
| Dear Nurse Workers:  We are researchers from xxx Hospital. We sincerely invite you to participate in our research project. This study aims to understand the nurses' Knowledge, Attitude, and Practice Toward the Sexual Health of Breast Cancer Patients, to serve as the basis for developing scientific intervention strategies, and may help many others in the future to improve their health conditions. Your participation in this study is voluntary, and the research has been approved by the Ethics Review Committee. If you agree to participate, please read the following instructions:   1. Please complete the questionnaire, the answer is not right or wrong, you only need to fill in according to the actual situation. You can ask us any questions in the process of answering, and please submit it in time after you finish it. 2. This study is a simple questionnaire survey, which will not harm your physical and psychological condition, but it will involve some privacy issues, such as your gender, age, etc. We will keep strict confidentiality and will not disclose your information, so please feel free to fill it out. 3. As a participant, you can keep yourself informed of the information related to this study and the progress of the study. If you decide to withdraw from the study, please let us know that your data will not be included in the results of the study.   Finally, we sincerely thank you for taking time out of your busy schedule to support our scientific research!  □I have been informed and agreed to the use of the collected data for scientific research.  Informed Consent Signature:  Date of participation: YYYY MM DD | | | |
| **Part I Basic Information** | | | |
| **1. Your gender:** | | a. Male | b. Female |
| **2. Your age: years** | | | |
| **3. Your education:** | | a. High school/secondary school  b. Associate Degree  c. Bachelor's Degree  d. Master's Degree  e. Doctorate | |
| **4. Nature of Employing Institution:** | | a. Public primary  b. Public secondary  c. Public tertiary  d. Private hospitals | |
| **5. Your position:** | | a. Nurse  b. Physician  c. Rehabilitation therapist | |
| **6. Your job title:** | | a. None  b. Junior  c. Intermediate  d. Associate  e. Senior | |
| **7. Department:** | | a. Oncology  b. General Surgery/Thyroid and Breast Surgery/Breast Surgery  c. Radiotherapy | |
| **8. Your clinical work experience (years):** | | a. Less than 1 year  b. ≥1 but <3 years  c. ≥3 but <5 years  d. ≥5 but <10 years  e. ≥10 years | |
| **9. Have you ever managed a breast cancer patient?** | | a. Yes  b. No | |
| **10. Have you received any training or lectures on sexual health for breast cancer patients?** | | a. Yes  b. No | |
| **11. How satisfied are you with your current job: ________** (please rate on a scale of 0-10, with 0 indicating very dissatisfied and 10 indicating very satisfied) | | | |

**Part II Knowledge of Sexual Health of Breast Cancer Patients**

| **K1. Sexual activity is not limited to sexual intercourse; it can also involve sensations, touch, and other ways to create intimacy and warmth.** | | | | | | | a. Unknown | | b. Partly known | | c. Well known | |
| --- | --- | --- | --- | --- | --- | --- | --- | --- | --- | --- | --- | --- |
| **K2. Research indicates that sexual dysfunction occurs in over 70% of breast cancer patients, with a significantly higher rate in mainland China compared to other regions.** | | | | | | | a. Unknown | | b. Partly known | | c. Well known | |
| **K3. Breast cancer treatments, endocrine therapy, body image issues, anxiety, and depression are factors impacting breast cancer patients' sexual health.** | | | | | | | a. Unknown | | b. Partly known | | c. Well known | |
| **K4. Healthy and moderate sexual activity contributes to the physical and mental recovery of breast cancer patients, but patients need to be reminded about contraception.** | | | | | | | a. Unknown | | b. Partly known | | c. Well known | |
| **K5. Physical barrier contraception methods are recommended for birth control in breast cancer patients; hormonal contraception should be avoided.** | | | | | | | a. Unknown | | b. Partly known | | c. Well known | |
| **K6. Non-hormonal therapies are the preferred choice for addressing sexual difficulties in breast cancer patients, including using 4% lidocaine solution, water-based lubricants, lubricating oils, and vitamin D and E suppositories, all proven effective in clinical studies.** | | | | | | | a. Unknown | | b. Partly known | | c. Well known | |
| **K7. Aromatase inhibitors and anti-estrogen drugs (e. g. , tamoxifen) can lead to side effects such as ovarian suppression, menopause, and sexual dysfunction, resulting in decreased libido, painful intercourse, and other issues.** | | | | | | | a. Unknown | | b. Partly known | | c. Well known | |
| **K8. Most sexual issues in breast cancer patients are psychological rather than organic in nature; they stem mainly from psychological factors of patients and their partners. Therefore, psychological intervention is a crucial part of caring for breast cancer patients' sexual health.** | | | | | | | a. Unknown | | b. Partly known | | c. Well known | |
| **K9. Research indicates that proper exercise, cognitive-behavioral therapy, mindfulness therapy, and sexual psychological counseling can improve sexual function in breast cancer patients.** | | | | | | | a. Unknown | | b. Partly known | | c. Well known | |
| **K10. The Female Sexual Function Index questionnaire (FSFI) is a widely used assessment tool for evaluating the sexual health of breast cancer patients.** | | | | | | | a. Unknown | | b. Partly known | | c. Well known | |
| **K11. Patients' sexual lives are influenced by their partner's or sexual companion's attitude towards sex, so partners should also be included in the assessment to comprehensively evaluate patients' sexual quality of life.** | | | | | | | a. Unknown | | b. Partly known | | c. Well known | |
| **Part III Attitude to Sexual Health of Breast Cancer Patients** | | | | | | | | | | |  |  |
| **A1. Importance of sexual health for overall human health** | | a. Strongly Agree | | b. Agree | | c. Neutral | d. Disagree | | e. Strongly Disagree | |  |  |
| **A2. Sexual health is linked to morals and personal beliefs, should be positively guided at the societal level** | | a. Strongly Agree | | b. Agree | | c. Neutral | d. Disagree | | e. Strongly Disagree | |  |  |
| **A3. Influence of sexual health on the quality of life for breast cancer survivors** | | a. Strongly Agree | | b. Agree | | c. Neutral | d. Disagree | | e. Strongly Disagree | |  |  |
| **A4. Need for healthcare providers to address sexual health during follow-up visits for breast cancer patients** | | a. Strongly Agree | | b. Agree | | c. Neutral | d. Disagree | | e. Strongly Disagree | |  |  |
| **A5. Appropriateness of using interviews, questionnaires, etc. , to assess sexual health in follow-up visits for breast cancer patients** | | a. Strongly Agree | | b. Agree | | c. Neutral | d. Disagree | | e. Strongly Disagree | |  |  |
| **A6. Importance of promoting knowledge about sexual health after breast cancer treatment to help patients understand and cope better** | | a. Strongly Agree | | b. Agree | | c. Neutral | d. Disagree | | e. Strongly Disagree | |  |  |
| **A7. Belief that sexual behavior is private and breast cancer patients might be sensitive to the topic, so healthcare providers shouldn't bring it up** | | a. Strongly Agree | | b. Agree | | c. Neutral | d. Disagree | | e. Strongly Disagree | |  |  |
| **A8. Feeling of discomfort or difficulty in addressing sexual health management questions with breast cancer patients** | | a. Strongly Agree | | b. Agree | | c. Neutral | d. Disagree | | e. Strongly Disagree | |  |  |
| **A9. Belief that sexual health issues aren't life-threatening and aren't within the scope of healthcare providers' responsibilities** | | a. Strongly Agree | | b. Agree | | c. Neutral | d. Disagree | | e. Strongly Disagree | |  |  |
| **A10. Perception of overall attention given by healthcare providers to sexual health of breast cancer patients** | | a. Strongly Agree | | b. Agree | | c. Neutral | d. Disagree | | e. Strongly Disagree | |  |  |
| **Part IV Practice on Sexual Health of Breast Cancer Patients** | | | | | | | | | | |  |  |
| **P1. During follow-up visits for breast cancer patients, you assess their sexual health using interviews, questionnaires, etc.** | a. Always | | b. Often | | c. Sometimes | | d. Occasionally | | e. Never | |  |  |
| **P2. Frequency of providing sexual health education to breast cancer patients during follow-up visits** | a. Always | | b. Often | | c. Sometimes | | d. Occasionally | | e. Never | |  |  |
| **P3. When providing treatment plans, you inform patients about potential impacts on sexual function and offer advice and treatment options** | a. Very consistent | | b. Consistent | | c. Neutral | | d. Less consistent | | e. Very inconsistent | |  |  |
| **P4. For breast cancer patients with postoperative breast loss, you provide relevant education about breast reconstruction before or after surgery** | a. Very consistent | | b. Consistent | | c. Neutral | | d. Less consistent | | e. Very inconsistent | |  |  |
| **P5. If patients don't bring up sexual health issues, you don't initiate discussions about them** | a. Very consistent | | b. Consistent | | c. Neutral | | d. Less consistent | | e. Very inconsistent | |  |  |
| **P6. In clinical practice, when assessing or educating about sexual health issues, you consider involving spouses and provide joint guidance** | a. Very consistent | | b. Consistent | | c. Neutral | | d. Less consistent | | e. Very inconsistent | |  |  |
| **P7. In clinical practice, you incorporate patients' sexual health issues into their individual treatment plans** | a. Very consistent | | b. Consistent | | c. Neutral | | d. Less consistent | | e. Very inconsistent | |  |  |
| **P8. You actively stay updated on research progress related to breast cancer patients' sexual health** | a. Very consistent | | b. Consistent | | c. Neutral | | d. Less consistent | | e. Very inconsistent | |  |  |
